# Surgical resection for second primary colorectal cancer: a population-based study

**DOI:** 10.3389/fmed.2023.1167777

**Published:** 2023-06-22

**Authors:** Ting Li, Zhenyang Liu, Fei Bai, Hua Xiao, Huijun Zhou

**Affiliations:** ^1^Department of Gastroenterology and Urology, Hunan Cancer Hospital, The Affiliated Cancer Hospital of Xiangya School of Medicine, Central South University, Changsha, Hunan, China; ^2^Department of Gastroduodenal Pancreas Surgery, Hunan Cancer Hospital, The Affiliated Cancer Hospital of Xiangya School of Medicine, Central South University, Changsha, Hunan, China; ^3^Department of Hepatobiliary and Intestinal Surgery, Hunan Cancer Hospital, The Affiliated Cancer Hospital of Xiangya School of Medicine, Central South University, Changsha, Hunan, China

**Keywords:** second primary cancer, colorectal cancer, surgical resection, segmental resection, outcome

## Abstract

**Background:**

Second primary colorectal cancer (CRC) is attributed to a crucial component of the CRC population. Still, its treatments remain unclear due to the troublesome conditions originating from multiple primary cancers and the lack of quality evidence. This study aimed to determine that which type of surgical resection is the eligible treatment for second primary CRC among patients with a prior cancer history.

**Methods:**

This cohort study retrospectively collected patients with second primary stage 0-III CRC in the Surveillance, Epidemiology, and End Results database from 2000 to 2017. Prevalence of surgical resection in second primary CRC, overall survival (OS) and disease-specific survival (DSS) of patients who received different surgical interventions were estimated.

**Results:**

A total of 38,669 patients with second primary CRC were identified. Most of the patients (93.2%) underwent surgical resection as initial treatment. Approximately 39.2% of the second primary CRCs (*N* = 15,139) were removed with segmental resection, while 54.0% (*N* = 20,884) were removed through radical colectomy/proctectomy. Surgical resection was associated with a significantly favorable OS and DSS compared to those not receiving any surgical operations for second primary CRC [OS: adjusted Hazard ratios (adjusted HR): 0.35; 95% CI: 0.34–0.37, *p* < 0.001; DSS: adjusted HR: 0.27; 95% CI: 0.25–0.29, *p* < 0.001]. Segmental resection considerably outperformed radical resection in terms of OS and DSS (OS: adjusted HR: 0.97; 95% CI: 0.91–1.00, *p* = 0.07; DSS: adjusted HR: 0.92; 95% CI: 0.87–0.97, *p* = 0.002). Segmental resection was also associated with a significantly reduced cumulative mortality of postoperative non-cancer comorbidities.

**Conclusion:**

Surgical resection demonstrated excellent oncological superiority for second primary CRC and was used to remove the vast majority of second primary CRCs. In comparison to radical resection, segmental resection offered a better prognosis and reduced postoperative non-cancer complications. The second primary colorectal cancers should be resected if the patients can afford surgical operations.

## Introduction

Colorectal cancer (CRC) is the third most common cancer in the world, accounting for 10.0% of incident cancer cases and 9.4% of incident cancer deaths in 2020 (1). CRC remains a severe health problem, especially in developed countries, where many older men in the general population suffer from this disease. In countries with high human development index, in 2020, the incidence rate of CRC was 29.0/100,000 males and 20.0/100,000 females, which were significantly higher than those with low human development index (1). For example, in the United States, by 2021, CRC is estimated to account for 149,500 new cancer cases and 52,980 new cancer deaths (2).

Any time a person has had one cancer, they can develop a second, new cancer unrelated to the prior one called a second primary cancer (SPC). It has been reported that the percentage of SPC in total cancer varies from 0.73 to 11.70% (3). Recent studies have shown that the incidence rate and risk of the second primary cancer are increasing (4, 5), which may be due to the extension of the life expectancy of cancer patients. Second primary CRC is one of the most common SPCs (4). It is important to study this special patient population (6).

There remain plenty of challenges regarding the treatments of patients with SPC or multiple primary cancers (MPCs) (7). When a patient has been diagnosed with two active malignant tumors at the same time, the challenge is to find an anti-cancer treatment strategy that covers both types of cancer, does not increase toxicity or related drug interactions, and does not have a negative impact on the overall results (8). For patients with a history of cancer and potential anti-cancer treatment, it may be difficult to determine the diagnosis of other primary tumors, because, for example, new metastasis may develop from the first cancer diagnosis, but it may also be part of the second malignant tumor (9). MPCs will also affect the registration of clinical research programs, because in most clinical trials, patients with previous cancer history or active secondary malignant tumors are usually excluded (10). Thus, high-quality evidence for the treatments of SPCs remained lacking.

Radical resection is the gold standard for first primary CRC, but when this is attributed to the troublesome conditions of SPCs, decision-making will be difficult. There is the possibility of prevention within this subset of second CRC, proposing different prophylactic actions such as extensive surgery or chemopreventive treatment (11, 12). Prophylactic resection of the entire colon has been identified as a preventive intervention for patients with familial adenomatous polyposis, which has the potency to develop into MPC (13). Although preventive surgery has been widely recognized, whether surgical resection is an eligible treatment for patients with second primary CRC is an unsolvable challenge.

The purpose of this study is to use population-based data sets to explore the prevalence and outcomes of surgical resection for second primary CRC among patients with a prior cancer history, and to investigate which type of surgical resection is the eligible treatment for second primary CRC.

## Materials and methods

### Data sources and study population

The Surveillance, Epidemiology and End Results (SEER) program provided the data for this backward, population-based, real-world study. The SEER database is a population-based cancer registry that collects information on cancer demographics, incidence rates, survival rates, and treatments (14). It covers about 30% of the US population. The research data protocol table must be signed in order to access the SEER database, which is open to the public. This study adhered to the recommendations of the STROCSS report (15).

We included all eligible patients diagnosed with the malignant second primary CRC (site code: C18.0, C18.2-C18.7, C19.9, C20.9) from 2000 to 2017. The third or above diagnosis of primary CRC was excluded. This study excluded patients whose diagnoses were made solely from autopsy or death certificate. We also excluded the patients without a microscopic confirmation. Patients were also omitted if they lacked comprehensive follow-up data, such as follow-up duration and age at diagnosis. Patients younger than 40 were also disqualified due to the low incidence rate of CRC. This study included patients with Stage 0-III CRC in order to precisely assess the surgical effect. Patients with stage IV CRC or with unknown stage information were excluded. Appendix tumor patients are not included (site code: C18.1). All practicable cases undergoing surgical resection were included, while the control group consisted of cases not undergoing any surgical resection. Using the patient ID provided by the SEER program, we further matched the information of second primary CRC with the prior tumor record. Those without definite information for prior cancer history were excluded (Supplementary Figure S1).

### Definition of variables

All patients were tracked from the time of their second primary diagnosis of CRC until they passed away, withdrew from the study while still alive, or the study came to an end (December 31, 2017). The following factors were examined among the study’s participants: age at diagnosis, gender, race, year of diagnosis, cancer stage, TNM stage as determined by the American Joint Commission on Cancer Staging (AJCC), surgical treatment, histological grading, urban/rural residency at diagnosis, median family income, follow-up duration, and life status at the conclusion of follow-up.

According to anatomical location, second primary CRCs were separated into proximal colon cancer (PCC), distal colon cancer (DCC), and rectal cancer (RC). PCCs include hepatic flexure cancer (site code: C18.3), transverse colon cancer (site code: C18.3), blind cancer (site code: C18.0), and ascending colon cancer (site code: C18.2) (site code: C18.4). DCC contains sigmoid colon cancer, descending colon cancer, and splenic flexure carcinoma (site codes: C18.5, C18.6) (site code: C18.7). RC comprises rectal cancer and rectal sigmoid junction cancer (site code: C19.9) (site code: C20.9).

The AJCC stage III code for patients diagnosed between 2000 and 2003, the ACCC stage VI code for patients diagnosed between 2004 and 2009, the AJCC stage VII code for patients diagnosed between 2010 and 2015, and the SEER joint staging for patients diagnosed between 2016 and 2017 are the sources for the TNM staging information (16).

Regarding individuals who are enrolled, the SEER program provides thorough site-specific surgery information (17, 18). The SEER program provides detailed information of the surgical treatment, including endoscopic destruction, endoscopic excision, segmental resection, and radical resection. In this study, we selected segmental resection and radical resection for investigation, as they were the two most prevalent surgical interventions for CRC (19–21). In surgical oncology, radical surgery refers to extensive and often mutilating surgery designed to remove all the diseased tissue (22). Segmental resection refers to the procedure removing the cancerous section of the colorectum, along with a small margin of healthy tissue, before the normal tissue is reconnected (23). For CRC, radical resection refers to colectomy for colon cancer or proctectomy for rectal cancer. For colon cancer, radical resection included subtotal/total colectomy, total proctocolectomy, and extended colectomy or proctocolectomy. For rectal cancer, radical resection included subtotal/total proctectomy, total proctocolectomy, and extended proctectomy or proctocolectomy. Segmental resection of CRC refers to the surgery to remove part of the colon/rectum to remove the tumor and normal tissue around it.

We gathered data on patients’ median family income and residence region (urban/rural) at the time of diagnosis in order to evaluate their socioeconomic position. The majority of these estimates are direct estimates of the characteristics of the target population in the survey sample data and are based on the population and socioeconomic level of a county.

Two categories were used to categorize the causes of death in second primary CRC patients: cancer deaths (i.e., CRC) and non-cancer comorbidities (i.e., deaths from any medical cause other than cancer) (18). The SEER cause-specific death classification variable from death certificates was used to identify the causes of death. Twenty six major groupings were used to classify non-cancer causes. These groupings were further divided into seven main categories: external injuries, other non-cancer causes, cardiovascular diseases (CVDs), respiratory diseases, gastrointestinal and liver diseases, and renal diseases (24).

### Analytical statistics

We compared the features of patients undergoing surgical resection with those of CRC patients who had not received any surgical procedures. We examined the patient population’s overall survival rate (OS) and disease-specific survival rate (DSS) using the Kaplan Meier method. The percentage of survivors (all-cause deaths) after follow-up is known as the OS rate. The percentage of patients who did not pass away from CRC (other than other causes) during a given time frame is known as the DSS rate (25). To assess the importance of the distinction between OS and DSS analysis, a COX regression model is used. For the groups with remarkable disparity in sample sizes, we adopted the Propensity Score Match (PSM) method to select 1:1 matched cases in the two group to avoid selection bias. The COX regression model was employed for OS and DSS analysis in order to further reduce the impact of baseline parameters, tumor features, and socioeconomic patient variables. Age, gender, race, the year of the diagnosis, the median family income, the patient’s urban or rural residency at the time of the diagnosis, the tumor grade, stage, and location were all taken into account while adjusting the model. Using the Kaplan–Meier approach, cumulative mortality rates (CMRs) for non-cancer comorbidities were calculated (18, 24). Versions 8.3.8 of SEER * Stat and R 3.6.3 were used for all analyses (14). The two-tailed test was used, and the statistically significant value of p was less than 0.05.

## Results

### Baseline characteristics

**Figure 1:**
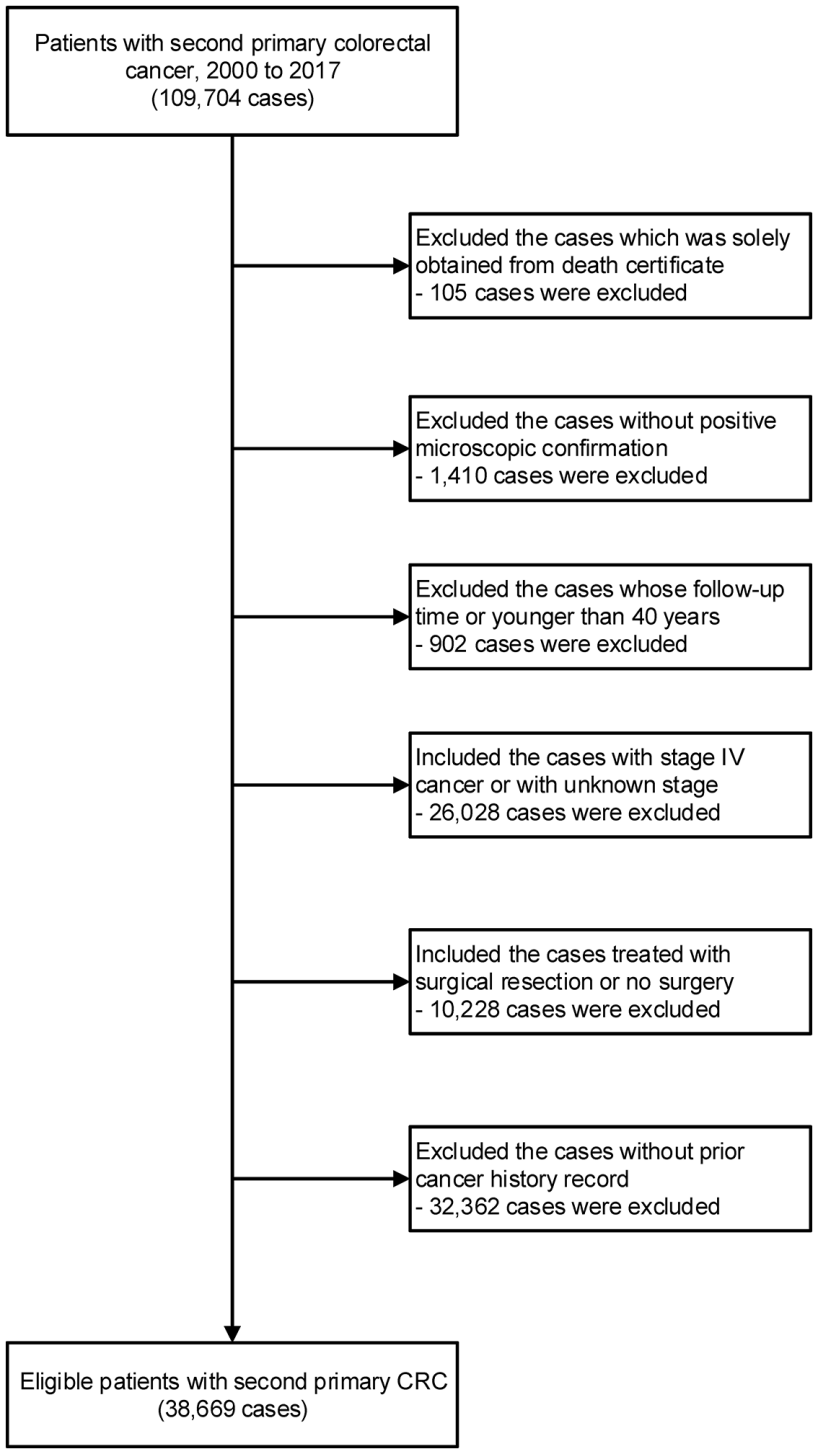
Inclusion and exclusion criteria for patients with second primary CRC in this study.

Among 38,669 patients with second primary stage 0-III CRC after a prior cancer history, 36,023 (93.2%) received surgical resection for the newly attained tumor (Table 1 and Figure 1). 3,053 cases (7.9%) of stage 0 CRC, 12,325 cases (31.9%) of stage I CRC, 12,239 cases (31.7%) of stage II CRC, and 11,052 cases (28.6%) of stage III CRC were included in this investigation. The proximal colon held 51.7% of the second primary CRC, the distal colon 26.0%, and the rectum 22.3% of the second primary CRC. The majority of the participating patients, who had second primary CRC, were between the ages of 60 and 79 (57.2%), were white (82.3%), non-Hispanic (92.2%), resided in metropolitan areas (86.6%), and came from families with median incomes (69.8%) (Table 1).

**Table 1:** Characteristics of patients with second primary CRC from 2000 to 2017.

Variable	No. of patients (%)	No. of deaths (%)
Total	38,669 (100%)	19,592 (100%)
Age		
40–59	5,834 (15.1%)	1,776 (9.1%)
60–79	22,117 (57.2%)	10,468 (53.4%)
80+	10,718 (27.7%)	7,348 (37.5%)
Sex		
Female	16,904 (43.7%)	8,267 (42.2%)
Male	21,765 (56.3%)	11,325 (57.8%)
Race		
White	31,836 (82.3%)	16,364 (83.5%)
Black	4,344 (11.2%)	2,220 (11.3%)
AI/AN	205 (0.5%)	90 (0.5%)
API	2,245 (5.8%)	911 (4.6%)
Unknown	39 (0.1%)	7 (0%)
Hispanic origin		
Non-Hispanic	35,670 (92.2%)	18,268 (93.2%)
Hispanic	2,999 (7.8%)	1,324 (6.8%)
Year of diagnosis		
2000–2004	6,197 (16%)	4,848 (24.7%)
2005–2009	11,490 (29.7%)	7,485 (38.2%)
2010–2017	20,982 (54.3%)	7,259 (37.1%)
Urban/rural residence		
Urban	33,485 (86.6%)	16,810 (85.8%)
Rural	5,121 (13.2%)	2,749 (14%)
Unknown	63 (0.2%)	33 (0.2%)
Median house-hold income		
Low	761 (2%)	383 (2%)
Median	26,998 (69.8%)	13,772 (70.3%)
High	10,909 (28.2%)	5,437 (27.8%)
Unknown	1 (0%)	0 (0%)
AJCC stage		
Stage 0	3,053 (7.9%)	1,527 (7.8%)
Stage I	12,325 (31.9%)	5,662 (28.9%)
Stage II	12,239 (31.7%)	6,368 (32.5%)
Stage III	11,052 (28.6%)	6,035 (30.8%)
Grade		
Grade I/II	28,105 (72.7%)	13,713 (70%)
Grade III/IV	6,230 (16.1%)	3,587 (18.3%)
Unknown	4,334 (11.2%)	2,292 (11.7%)
Anatomic sites		
PCC	19,994 (51.7%)	10,088 (51.5%)
Cecum	7,340 (19%)	3,701 (18.9%)
Ascending colon	7,013 (18.1%)	3,497 (17.8%)
Hepatic flexure	1,766 (4.6%)	901 (4.6%)
Transverse colon	3,875 (10%)	1,989 (10.2%)
DCC	10,036 (26%)	5,111 (26.1%)
Splenic flexure	1,138 (2.9%)	613 (3.1%)
Descending colon	2,163 (5.6%)	1,059 (5.4%)
Sigmoid colon	6,735 (17.4%)	3,439 (17.6%)
RC	8,639 (22.3%)	4,393 (22.4%)
Rectosigmoid junction	2,532 (6.5%)	1,299 (6.6%)
Rectum	6,107 (15.8%)	3,094 (15.8%)
Surgery type		
None	2,646 (6.8%)	1,842 (9.4%)
Radical surgery	20,884 (54%)	10,520 (53.7%)
Segmental resection	15,139 (39.2%)	7,230 (36.9%)
Reason no surgery		
Surgery performed	36,023 (93.2%)	17,750 (90.6%)
Not performed, patient died	27 (0.1%)	27 (0.1%)
Not recommended, contraindicated	224 (0.6%)	186 (0.9%)
Not recommended	1,812 (4.7%)	1,242 (6.3%)
Recommended, patient refused	238 (0.6%)	171 (0.9%)
Recommended but not performed, unknown reason	223 (0.6%)	155 (0.8%)
Temporality of second cancer^1^		
Synchronous	9,777 (25.3%)	5,747 (29.3%)
Metachronous	28,892 (74.7%)	13,845 (70.7%)
Prior cancer type		
CRC	13,340 (34.5%)	7,290 (37.2%)
Non-CRC	25,329 (65.5%)	12,302 (62.8%)
Prostate	7,632 (19.7%)	3,626 (18.5%)
Breast	4,956 (12.8%)	1,984 (10.1%)
Urinary system	2,307 (6%)	1,206 (6.2%)
Non-basal Skin	1,812 (4.7%)	755 (3.9%)
Lung and Bronchus	1,541 (4%)	1,108 (5.7%)
Lymphoma	1,222 (3.2%)	646 (3.3%)
Corpus uterus	1,141 (3%)	422 (2.2%)
Other	4,718 (12.2%)	2,555 (13.0%)

AI/AN, American Indian/Alaska Native; API, Asian or Pacific Islander; PCC, proximal colon cancer; DCC, distal colon cancer; RC, rectal cancer.

^1^Synchronous second primary cancers refer to those diagnosed within 2 months since the prior cancer diagnosis.

Of the second primary CRCs, approximately 39.2% (*N* = 15,139) were removed with segmental resection, while the remaining 54.0% (*N* = 20,884) were removed through radical resection (Table 1). Given that radical resection was used to treat more than 75% of tumors in the cecum, ascending colon, and hepatic flexure, tumors in the proximal colon are more likely to be removed by this method. The segmental resection will be a more common alternative (> 50%) for those situated in the distal site, such as the sigmoid colon and rectum (Supplementary Figure S1A). The elderly patients with second primary CRC were more likely to undergo radical resection than segmental resection (Supplementary Figure S1B).

### Survival analysis of surgical resection for second primary CRC

**Figure 2:**
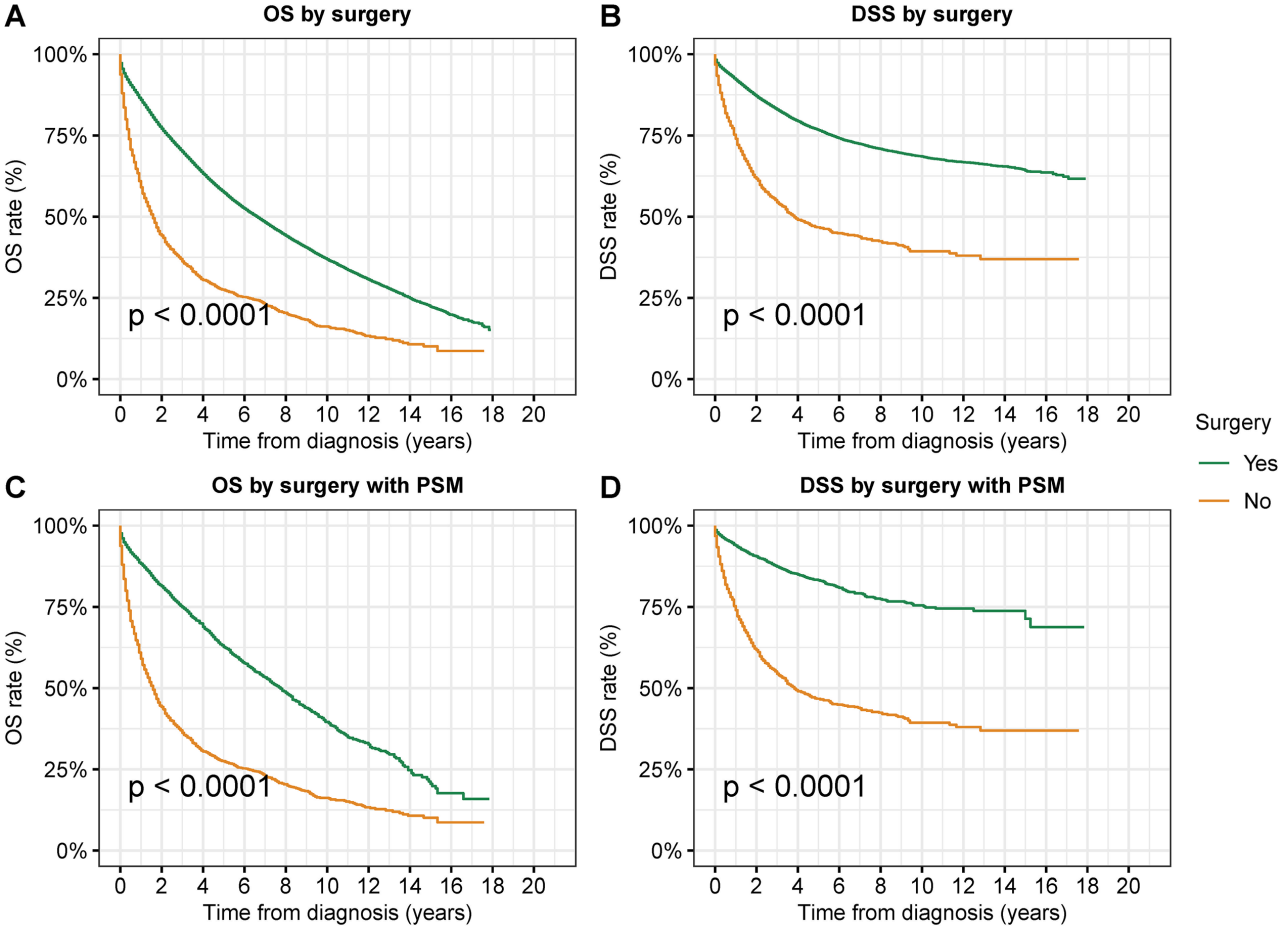
Overall survival (OS) and disease-specific survival (DSS) of patients with second primary CRC by surgical resection. **(A)** OS and **(B)** DSS of patients with second primary CRC by surgical resection. **(C)** OS and **(D)** DSS of patients with second primary CRC by surgical resection after PSM.

To research the effectiveness of surgical resection in the treatment of second primary CRC, we performed survival analyses of surgical resection using those who underwent no surgical treatments as the control group (Figure 2 and Supplementary Figures S2, S3). In patients with second primary CRC, surgical resection significantly improved both OS and DSS (OS: 57.5% vs. 27.5% at 5 years, *p* < 0.001; DSS: 76.6% vs. 46.7% at 5 years, *p* < 0.001) (Figures 2A,B). As there was remarkable disparity in sample sizes between these two group, we used PSM matching to select 1:1 matched patients in these two groups. After PSM by variables including age, sex, race, year of diagnosis, median income, rural/urban residence, AJCC T stage, and AJCC N stage, 2,645 cases with surgical resection and 2,645 cases with no surgery had been selected. After PSM, surgical resection was also related with significantly improved both OS and DSS (Figures 2C,D). This benefit of surgical resection for survival was frequently observed in CRC patients of all stages (OS: **stage 0**: 69.8% vs. 35.5% at 5 years, *p* < 0.001; **stage I**: 66.9% vs. 25.5% at 5 years, *p* < 0.001; **stage II**: 55.2% vs. 23.7% at 5 years, p < 0.001; stage III: 46.9% vs. 20.2% at 5 years, *p* < 0.001) (Supplementary Figure S2).

In the multivariate Cox analyses adjusted by age, race, gender, year of diagnosis, residential area, household income, tumor site, tumor grade, AJCC stage, prior cancer type, and temporality of second cancer to compare the prognosis after surgery, surgical resection was associated with a significantly favorable OS [adjusted Hazard ratios (adjusted HR): 0.35; 95% CI: 0.34–0.37, *p* < 0.001] and DSS (adjusted HR: 0.27; 95% CI: 0.25–0.29, *p* < 0.001) compared to those not receiving any surgical interventions (Table 2).

**Table 2:** Multivariable COX analysis of the overall survival and disease-specific survival by surgery of patients diagnosed with second primary colorectal cancer.

Variables	Overall survival		Cancer-specific survival	
	HR (95% CI)	*p*	HR (95% CI)	*p*
Age				
40–59	Reference		Reference	
60–79	1.88 (1.79–1.98)	**<0.001**	1.37 (1.29–1.47)	**0.002**
80+	3.96 (3.76–4.18)	**<0.001**	2.45 (2.28–2.63)	**<0.001**
Sex				
Female	Reference		Reference	
Male	1.13 (1.1–1.16)	**<0.001**	1.10 (1.05–1.14)	**<0.001**
Race				
White	Reference		Reference	
Black	1.14 (1.09–1.19)	**<0.001**	1.18 (1.11–1.26)	**<0.001**
AI/AN	0.96 (0.74–1.24)	0.7	0.92 (0.63–1.34)	0.7
API	0.81 (0.76–0.87)	**<0.001**	0.89 (0.81–0.98)	**0.02**
Unknown	0.36 (0.17–0.75)	**0.007**	0.22 (0.05–0.86)	**0.03**
Year of diagnosis				
2000–2004	Reference		Reference	
2005–2009	0.97 (0.93–1.00)	0.08	0.95 (0.89–1.00)	**0.07**
2010–2017	0.88 (0.84–0.92)	**<0.001**	0.86 (0.81–0.91)	**<0.001**
Median house-hold income				
Low	1.20 (1.07–1.34)	**0.002**	1.02 (0.86–1.21)	0.8
Median	1.06 (1.03–1.09)	**0.001**	1.10 (1.05–1.16)	**<0.001**
High	Reference		Reference	
Unknown	–	1	–	1
Residence region				
Rural	Reference		Reference	
Urban	0.93 (0.89–0.97)	**0.002**	0.91 (0.85–0.97)	**0.003**
Unknown	1.38 (0.9–2.13)	0.1	1.66 (0.92–3.02)	0.1
Grade				
Grade I/II	Reference		Reference	
Grade III/IV	1.20 (1.16–1.25)	**<0.001**	1.39 (1.32–1.46)	**<0.001**
Unknown	1.03 (0.97–1.08)	0.3	1.03 (0.95–1.11)	0.5
Anatomic sites				
DCC	Reference		Reference	
PCC	0.96 (0.93–1)	**0.03**	0.91 (0.86–0.96)	**0.01**
RC	1.01 (0.97–1.05)	0.7	1.13 (1.07–1.20)	**<0.001**
AJCC T stage				
Stage 0	Reference		Reference	
Stage I	1.04 (1.01–1.09)	**0.001**	1.20 (1.08–1.34)	**0.001**
Stage II	1.41 (1.32–1.51)	**<0.001**	1.96 (1.75–2.18)	**<0.001**
Stage III	1.85 (1.73–1.97)	**<0.001**	3.48 (3.12–3.87)	**<0.001**
Prior cancer type				
CRC	Reference		Reference	
Non-CRC	1.02 (0.99–1.06)	0.3	0.67 (0.64–0.70)	**<0.001**
Temporality of second cancer				
Synchronous	1.21 (1.17–1.26)	**<0.001**	1.10 (1.04–1.16)	**0.001**
Metachronous	Reference		Reference	
Surgery				
Yes	0.35 (0.34–0.37)	**<0.001**	0.27 (0.25–0.29)	**<0.001**
No	Reference		Reference	

AI/AN, American Indian/Alaska Native; API, Asian or Pacific Islander; PCC, proximal colon cancer; DCC, distal colon cancer; RC, rectal cancer.

### Survival analysis of segmental resection and radical surgery for second primary CRC

**Figure 3:**
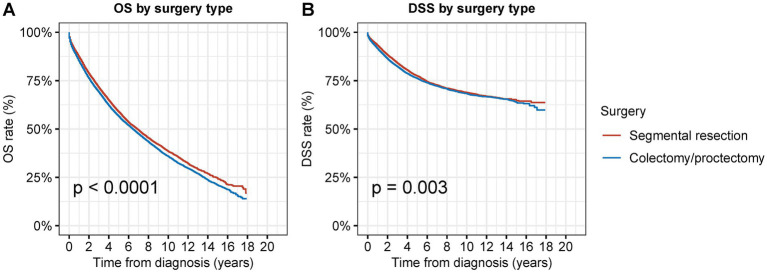
Overall survival (OS) and disease-specific survival (DSS) of patients with second primary CRC by types of surgical procedures. **(A)** OS and **(B)** DSS of patients with second primary CRC by types of surgical procedures.

**Figure 4:**
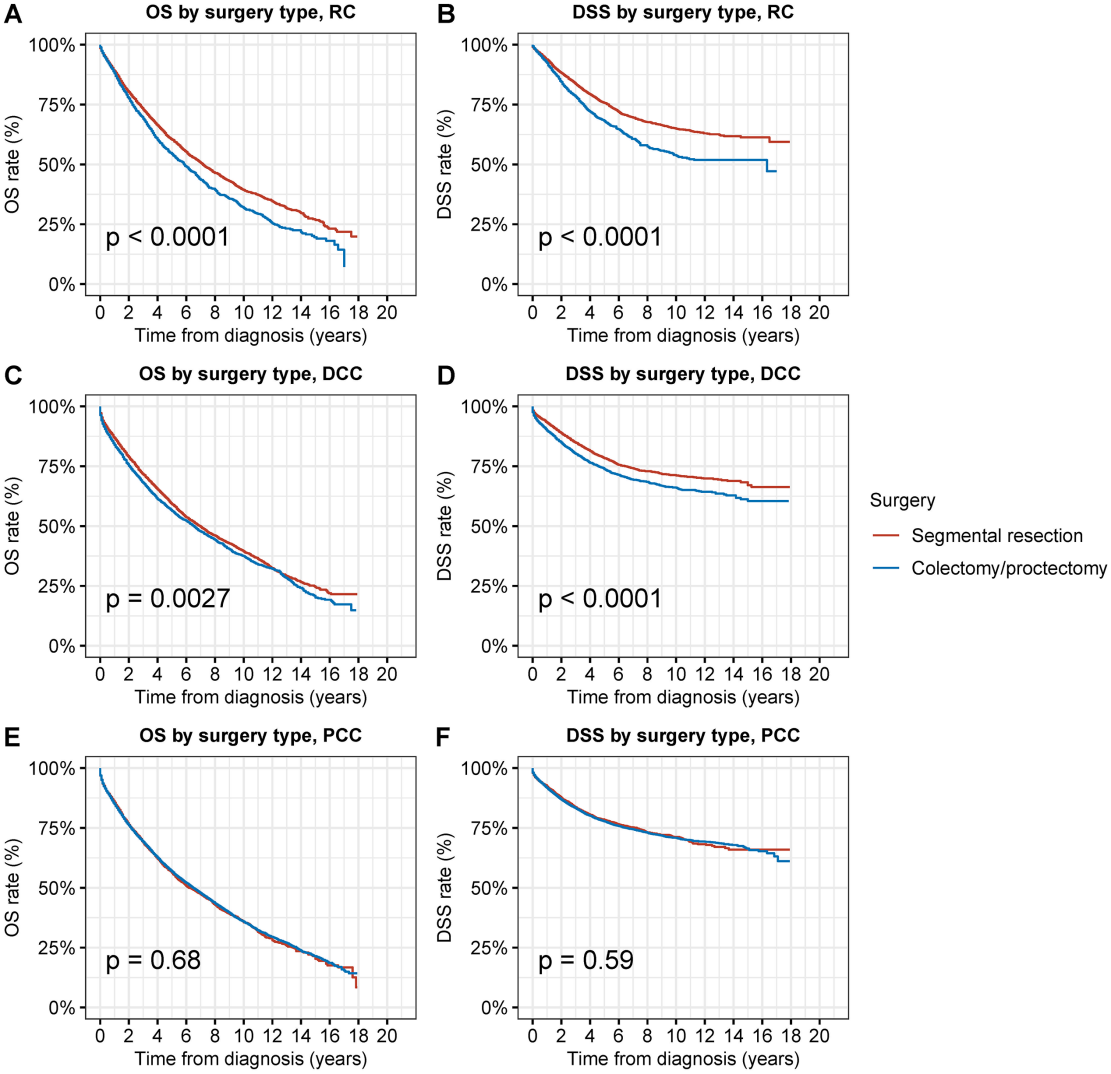
Overall survival (OS) and disease-specific survival (DSS) of patients with second primary CRC by types of surgical procedures and tumor sites. **(A)** OS and **(B)** DSS of patients with second primary RC by types of surgical procedures. **(C)** OS and **(D)** DSS of patients with second primary DCC by types of surgical procedures. **(E)** OS and **(F)** DSS of patients with second primary PCC by types of surgical procedures.

**Figure 5:**
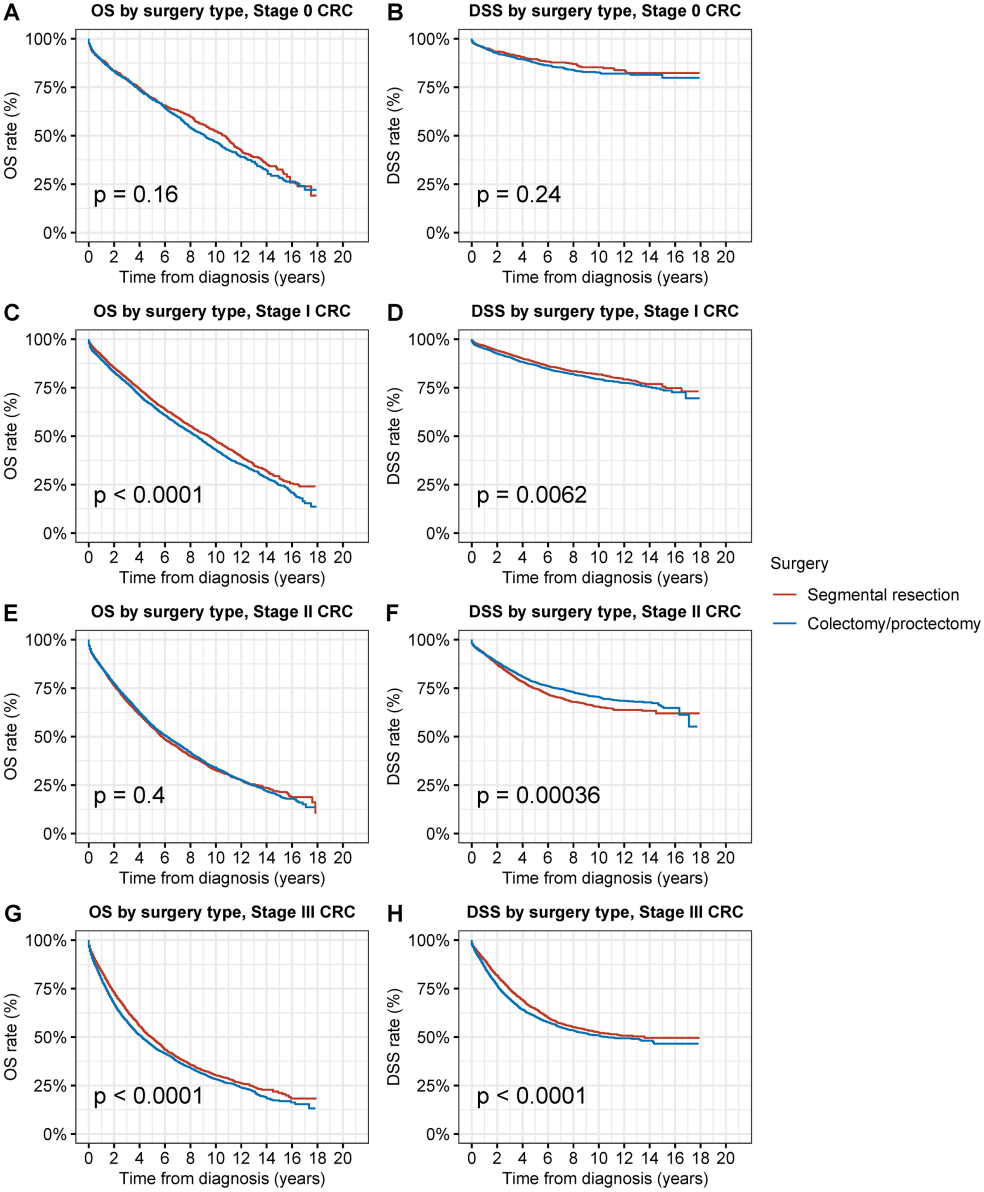
Overall survival (OS) and disease-specific survival (DSS) of patients with second primary CRC by types of surgical procedures and tumor stage. **(A)** OS and **(B)** DSS of patients with stage 0 s primary CRC by types of surgical procedures. **(C)** OS and **(D)** DSS of patients with stage I second primary CRC by types of surgical procedures. **(E)** OS and **(F)** DSS of patients with stage II second primary CRC by types of surgical procedures. **(G)** OS and **(H)** DSS of patients with stage III second primary CRC by types of surgical procedures.

We examined the prognosis following segmental resection and radical surgery among patients with second primary CRC in order to determine the superior surgical alternatives (Figures 3–5). Segmental resection considerably outperformed radical resection in terms of OS and DSS for all sites combined (OS: 58.8% vs. 56.5% at 5 years, *p* < 0.001; DSS: 77.4% vs. 76.1% at 5 years, *p* = 0.003) (Figure 3).

In the multivariate Cox analyses, segmental resection was associated with a slightly better OS (adjusted HR: 0.97; 95% CI: 0.91–1.00, *p* = 0.07) and a significantly better DSS (adjusted HR: 0.92; 95% CI: 0.87–0.97, *p* = 0.002) compared to those not receiving any surgical interventions (Table 3).

**Table 3:** Multivariable COX analysis of the overall survival and disease-specific survival by types of surgical intervention of patients diagnosed with second primary colorectal cancer.

Variables	Overall survival		Cancer-specific survival	
	HR (95% CI)	*p*	HR (95% CI)	*p*
Age				
40–59	Reference		Reference	
60–79	1.91 (1.81–2.02)	**<0.001**	1.39 (1.3–1.49)	**<0.001**
80+	4.09 (3.87–4.33)	**<0.001**	2.50 (2.32–2.7)	**<0.001**
Sex				
Female	Reference		Reference	
Male	1.14 (1.11–1.18)	**<0.001**	1.12 (1.07–1.17)	**<0.001**
Race				
White	Reference		Reference	
Black	1.14 (1.09–1.20)	**<0.001**	1.19 (1.11–1.28)	**<0.001**
AI/AN	1.00 (0.77–1.31)	1	1.01 (0.69–1.46)	1
API	0.80 (0.74–0.86)	**<0.001**	0.87 (0.79–0.97)	**0.009**
Unknown	0.39 (0.16–0.95)	**0.04**	0.15 (0.02–1.1)	0.06
Year of diagnosis				
2000–2004	Reference		Reference	
2005–2009	0.97 (0.93–1.01)	0.1	0.94 (0.88–1.00)	**0.04**
2010–2017	0.88 (0.84–0.92)	**<0.001**	0.86 (0.81–0.92)	**<0.001**
Median house-hold income				
Low	1.21 (1.07–1.36)	**0.002**	1.03 (0.85–1.25)	0.8
Median	1.07 (1.03–1.10)	**<0.001**	1.11 (1.05–1.17)	**<0.001**
High	Reference		Reference	
Residence region				
Rural	Reference		Reference	
Urban	0.94 (0.90–0.99)	0.4	0.93 (0.87–1.00)	0.05
Unknown	1.29 (0.82–2.02)	0.3	1.47 (0.79–2.72)	0.2
Grade				
Grade I/II	Reference		Reference	
Grade III/IV	1.19 (1.15–1.24)	**<0.001**	1.38 (1.31–1.46)	**<0.001**
Unknown	1.05 (0.99–1.12)	**0.1**	1.12 (1.01–1.23)	**0.03**
Anatomic sites				
DCC	Reference		Reference	
PCC	0.94 (0.91–0.98)	**0.003**	0.87 (0.82–0.92)	**<0.001**
RC	1.03 (0.99–1.08)	0.1	1.20 (1.12–1.28)	**<0.001**
AJCC stage				
Stage 0	Reference		Reference	
Stage I	1.07 (0.99–1.15)	0.07	1.22 (1.06–1.39)	**0.005**
Stage II	1.41 (1.31–1.52)	**<0.001**	2.16 (1.88–2.48)	**<0.001**
Stage III	1.86 (1.72–2.00)	**<0.001**	3.93 (3.43–4.5)	**<0.001**
Prior cancer type				
CRC	Reference		Reference	
Non-CRC	1.01 (0.97–1.05)	0.6	0.68 (0.64–0.72)	**<0.001**
Temporality of second cancer				
Synchronous	1.20 (1.15–1.25)	**<0.001**	1.10 (1.04–1.17)	**0.002**
Metachronous	Reference		Reference	
Surgery				
Segmental resection	0.97 (0.91–1.00)	**0.07**	0.92 (0.87–0.97)	**0.002**
Colectomy/proctectomy	Reference		Reference	

AI/AN, American Indian/Alaska Native; API, Asian or Pacific Islander; PCC, proximal colon cancer; DCC, distal colon cancer; RC, rectal cancer.

When compared to radical resection, segmental resection for second primary RC was associated with considerably superior OS and DSS (OS: 70.0% vs. 64.3% at 5 years, *p* < 0.001; DSS: 87.7% vs. 81.1% at 5 years, *p* < 0.001) (Figures 4A,B). Compared to radical resection, segmental resection for DCC was also associated with considerably superior OS and DSS (OS: 69.6% vs. 66.5% at 5 years, *p* = 0.02; DSS: 90.2% vs. 86.4% at 5 years, *p* < 0.001) (Figures 4C,D). In cases of PCC, segmental resection produced outcomes that were comparable to those of radical resection in terms of OS and DSS (OS: 66.1% vs. 67.2% at 5 years, *p* = 0.9; DSS: 90.0% vs. 89.8% at 5 years, *p* = 0.6) (Figures 4E,F).

### Survival analysis of segmental resection and radical surgery for second primary CRC by stage

Segmental resection demonstrated comparable OS and DSS compared to radical resection for stage 0 s primary CRC (OS: 69.4% vs. 68.6% at 5 years, *p* = 0.2; DSS: 89.5% vs. 87.5% at 5 years, *p* = 0.3) (Figures 5A,B). Segmental resection significantly improved OS and DSS compared to radical resection for stage I second primary CRC (OS: 68.4% vs. 65.7% at 5 years, *p* < 0.001; DSS: 88.0% vs. 86.6% at 5 years, *p* = 0.006) (Figures 5C,D). Segmental resection demonstrated comparable OS and worse DSS compared with radical resection for stage II second primary CRC (OS: 54.6% vs. 55.6% at 5 years, *p* = 0.4; DSS: 74.7% vs. 78.0% at 5 years, *p* < 0.001) (Figures 5E,F). Segmental resection demonstrated comparable OS and DSS compared with radical resection for stage III second primary CRC (OS: 49.2% vs. 45.4% at 5 years, *p* < 0.001; DSS: 64.3% vs. 60.4% at 5 years, *p* < 0.001) (Figures 5G,H). Segmental resection demonstrated considerably superior or equivalent OS and DSS compared with radical resection for the second primary CRC of all sites and all stages (Supplementary Figures S4–S6).

### Cause of death of surgical resection for second primary CRC

**Figure 6:**
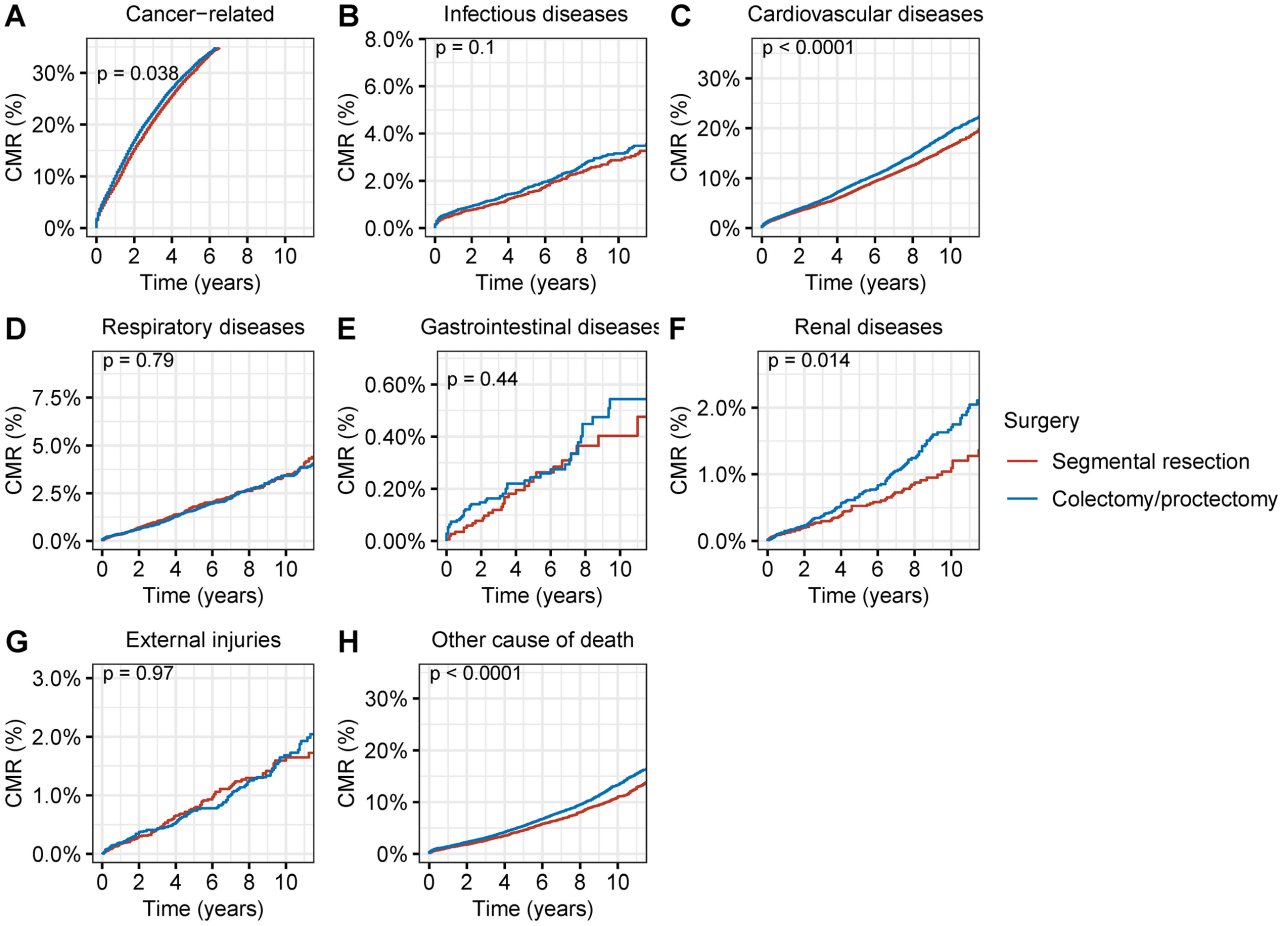
Cumulative mortality rate (CMR) among patients with second primary CRC by different types of surgical operation. **(A)** CMR from cancer-related deaths among patients with second primary CRC by different types of surgical operation. **(B)** CMR from infectious diseases among patients with second primary CRC by different types of surgical operation. **(C)** CMR from cardiovascular diseases among patients with second primary CRC by different types of surgical operation. **(D)** CMR from respiratory diseases among patients with second primary CRC by different types of surgical operation. **(E)** CMR from gastrointestinal diseases among patients with second primary CRC by different types of surgical operation. **(F)** CMR from renal diseases among patients with second primary CRC by different types of surgical operation. **(G)** CMR from external injuries among patients with second primary CRC by different types of surgical operation. **(H)** CMR from other non-cancer causes among patients with second primary CRC by different types of surgical operation.

Patients who received segmental resection as opposed to radical resection had a considerably decreased CMR of cancer-related fatalities in those with second primary CRC (*p* = 0.04) (Figure 6A). Patients who underwent segmental resection also had significantly lower CMR from CVDs (*p* < 0.001), renal diseases (*p* = 0.01), (*p* < 0.001), and other diseases (*p* < 0.001) (Figures 6B–H). A much reduced CMR of postoperative non-cancer comorbidities was linked to segmental resection in surgical procedures.

## Discussion

In this population-based analysis, we investigated the prevalence and therapeutic effectiveness of surgical resection for second primary CRC in more than 30,000 patients with stage 0-III disease. We discovered that surgical resection displayed great oncological superiority for second primary CRC and was used to remove the vast majority of second primary CRCs. Comparable outcomes and fewer postoperative non-cancer complications were offered by segmental resection compared to radical resection. These findings suggested that segmental resection, in particular, was beneficial for stage 0-III second primary CRC.

This study offers new proof for the use of surgical resection in people with second primary CRC, and serves as an excellent illustration in the area of healthcare of people with SPC or MPCs. Treatment of patients with MPCs is difficult and frequently involves a therapeutic conundrum (7, 8). There is currently little research available on how to treat patients with MPCs. On this unique event, there were only a few case reports or case series (26–28). Multidisciplinary team (MDT) meetings should be used to discuss patients with multiple primary, and it may require more than one MDT to reach an agreement on a treatment plan. The patient should also be made aware of the circumstances, therapeutic difficulties, and frequently the unpredictability of the prognosis since the therapy method needs to be modified. Only a few cancer types had recommendations to support the use of surgical excision for SPCs (29, 30). Our work will contribute to the body of knowledge supporting the use of surgical resection for CRC. More evidence is critically required for this condition since, in cases of advanced disease, choosing an antitumor therapy would be challenging and frequently not be supported by data from recent studies and the literature (7). Regarding the subject of treating patients with synchronous or metachronous MPCs, more investigation is required. Additionally, it’s important to better identify how previous therapy affected prognosis, antitumor effectiveness, and toxicity.

Our research proposes a fresh field for segmental resection in which it can be used safely. Less trauma, less bleeding, a shorter hospital stay, and simpler reconstruction are the major benefits of segmental resection. Due to a decreased median number of stools per day, individuals who received a (sub)total colectomy had a poorer quality of life than those who underwent a segmental resection (31). According to Bakker et al.’s analysis of 15,667 individuals who underwent resection for colon cancer, a subtotal colectomy was linked to a higher risk of anastomotic leakage but not to a higher risk of mortality (32). However, Klima et al. discovered that a subtotal colectomy increased the risk of mortality by 2.4 times (33). Kim and Park suggested that segmental resection should be carried out whenever practical because (sub)total colectomy has no benefit in terms of survival (34). According to Degiuli et al., segmental colonic resection is a safe and efficient therapeutic option for colon cancer of the splenic flexure (35). The segmental resection will offer patients with MPCs the special benefit of lower comorbidity rates and equivalent tumor clearance since extra care should be taken to minimize therapy-associated side effects in the complex scenarios created by MPCs and treatments for each individual tumor.

The most concerning issue of segmental resection, however, is that segmental resection might result in inadequate resection or a subsequent recurrence, which would constitute therapeutic failure and preclude the expansion of segmental resection (36, 37). The original lesion must be removed together with the accompanying lymphatic drainage area using the conventional oncologic strategy for colorectal cancer (38). Although segmental resection may be sufficient for the removal of the core tumor in order to address these issues, extended resections are typically necessary for a sufficient lymphadenectomy. Resection margins, both proximal and distal, must be at least 5 cm away from the tumor and must permit a sufficient resection of the affected intestinal segment, together with its vascular supply and lymphatic drainage. Total colectomy should be an option for high-risk individuals, such as those with Lynch syndrome, due to the increased risk of developing further metachronous CRC (39). After segmental resection, the odds of metachronous tumors are reported to be 16% at 10 years, 41% at 20 years, and 62% at 30 years (12, 40). Patients must be informed of their potential for metachronous tumors and the existing options, which include periodic colonoscopic surveillance (41).

This study had several limitations. We were unable to prospectively evaluate the impact of surgical therapies in patients with second main CRC and make causal inferences due to the descriptive and retrospective study design. Second, we were unable to evaluate the patient’s physical status, comorbidities, and other aspects of their health. It is crucial to consider these aspects when suggesting treatment options due to the high prevalence of comorbidities, cognitive impairment, frailty, functional losses, social isolation, and other problems in this population, but the SEER program did not give this information. Third, we were unable to examine the impact of additional treatments such as radiation, chemotherapy, and other targeted therapies. Only surgical procedures were covered in detail by the SEER study. Finally, this study spanned 17 years, many changes would happen in this period, and due to the delay of research data, we could not include the most recent years.

Despite these drawbacks, this study might have a significant impact on the literature on surgical treatments and cancer surveillance for second primary CRC. Radical resection is the gold standard for first primary CRC, but it is difficult to decide the treatments when this is attributed to the troublesome conditions of SPCs (7, 8). The previous literature has well described the preventive surgery of second primary CRC (11–13). Although surgical resection might be a routine procedure in the clinical practice for second primary CRC, there turns to be nearly no evidence on whether surgical resection is an eligible treatment for patients with second primary CRC. This population-based analysis was the first study to investigate the prevalence and therapeutic effectiveness of surgical resection for second primary CRC in more than 30,000 patients with stage 0-III disease. We discovered that surgical resection displayed great oncological superiority for second primary CRC and was used to remove the vast majority of second primary CRCs. Comparable outcomes and fewer postoperative non-cancer complications were offered by segmental resection compared to radical resection. Our results suggested that segmental resection was beneficial for stage 0-III second primary CRC. The data used in this investigation were obtained from a reliable, population-based, real-world cancer registry, which is its key strength. The results of this study have crucial ramifications for the growth of CRC. With such a large patient population from many different centers, probably there is not a uniform definition for segmental and radical resection. There is no data on type of colectomy/proctectomy and on lymph node clearance performed in different hospitals. Great caution should be used before suggesting that segmental resection is better than radical resection for colorectal tumors of any anatomical site. More evidence was needed to support the finding of our study.

## Conclusion

In conclusion, the vast majority of second primary CRCs were resected via surgical operations, and surgical resection exhibited excellent oncological superiority for second primary CRC. Segmental resection provided a comparable prognosis and fewer post-operation comorbidities than radical resection. The second primary CRCs should be resected if the patients’ health conditions can afford surgical operations. This study provides evidence to support the applications of surgical resection in patients with second primary CRC, and provides a good example for the treatments of patients with other SPC or MPCs. Further studies are needed to explore viable treatments for MPCs.

## Data availability statement

Publicly available datasets were analyzed in this study. This data can be found here: All data used in this study can be freely accessed from the SEER program (https://seer.cancer.gov/).

## Author contributions

TL: methodology, validation, data curation, resources, investigation, writing—original draft, and writing—review and editing. ZL: resources, formal analysis, visualization, and writing—review and editing. FB: methodology and writing—review and editing. HX: data curation, resources, methodology, and writing—review and editing. HZ: conceptualization, validation, and writing—review and editing, supervision, project administration, funding acquisition. All authors contributed to the article and approved the submitted version.

## Funding

This study was supported by the National Natural Science Foundation of China (No. 82203499); Hunan Provincial Natural Science Foundation of China (No. 2023JJ40409) and Hunan Cancer Hospital Climb Plan (No. 2021NSFC-B004). The funding sources had no involvement in study design; collection, management, analysis and interpretation of data; or the decision to submit for publication.

## Conflict of interest

The authors declare that the research was conducted in the absence of any commercial or financial relationships that could be construed as a potential conflict of interest.

The reviewer YZ declared a shared affiliation, with no collaboration, with one of the authors TL to the handling editor at the time of the review.

## Publisher’s note

All claims expressed in this article are solely those of the authors and do not necessarily represent those of their affiliated organizations, or those of the publisher, the editors and the reviewers. Any product that may be evaluated in this article, or claim that may be made by its manufacturer, is not guaranteed or endorsed by the publisher.
