# Supplementary material for: Surgical resection for second primary colorectal cancer: a population-based study
Source: Front Med (Lausanne). 2023 Jun 22;10:1167777. doi: 10.3389/fmed.2023.1167777 (PMC10324975; doi:10.3389/fmed.2023.1167777)
Supplement: Supplementary file 1 [file Data_Sheet_1.DOCX]

**Supplementary Figure Legend**

**Figure S1.** Trends of surgical rates by anatomic sites and age. (A) Trends of surgical rates by anatomic sites. (B) Trends of surgical rates by age.

**Figure S2.** Overall survival (OS) of patients with second primary CRC by surgery and tumor stage. OS of patients with (A) stage 0, (B) stage I, (C) stage II, (D) stage III second primary CRC by surgery.

**Figure S3.** Disease-specific survival (DSS) of patients with second primary CRC by surgery and tumor stage. DSS of patients with (A) stage 0, (B) stage I, (C) stage II, (D) stage III second primary CRC by surgery.

**Figure S4.** Overall survival (OS) and disease-specific survival (DSS) of patients with second primary RC by types of surgical procedures and tumor stage. (A) OS and (B) DSS of patients with stage 0 second primary RC by types of surgical procedures. (C) OS and (D) DSS of patients with stage I second primary RC by types of surgical procedures. (E) OS and (F) DSS of patients with stage II second primary RC by types of surgical procedures. (G) OS and (H) DSS of patients with stage III second primary RC by types of surgical procedures.

**Figure S5.** Overall survival (OS) and disease-specific survival (DSS) of patients with second primary DCC by types of surgical procedures and tumor stage. (A) OS and (B) DSS of patients with stage 0 second primary DCC by types of surgical procedures. (C) OS and (D) DSS of patients with stage I second primary DCC by types of surgical procedures. (E) OS and (F) DSS of patients with stage II second primary DCC by types of surgical procedures. (G) OS and (H) DSS of patients with stage III second primary DCC by types of surgical procedures.

**Figure S6.** Overall survival (OS) and disease-specific survival (DSS) of patients with second primary PCC by types of surgical procedures and tumor stage. (A) OS and (B) DSS of patients with stage 0 second primary PCC by types of surgical procedures. (C) OS and (D) DSS of patients with stage I second primary PCC by types of surgical procedures. (E) OS and (F) DSS of patients with stage II second primary PCC by types of surgical procedures. (G) OS and (H) DSS of patients with stage III second primary PCC by types of surgical procedures.


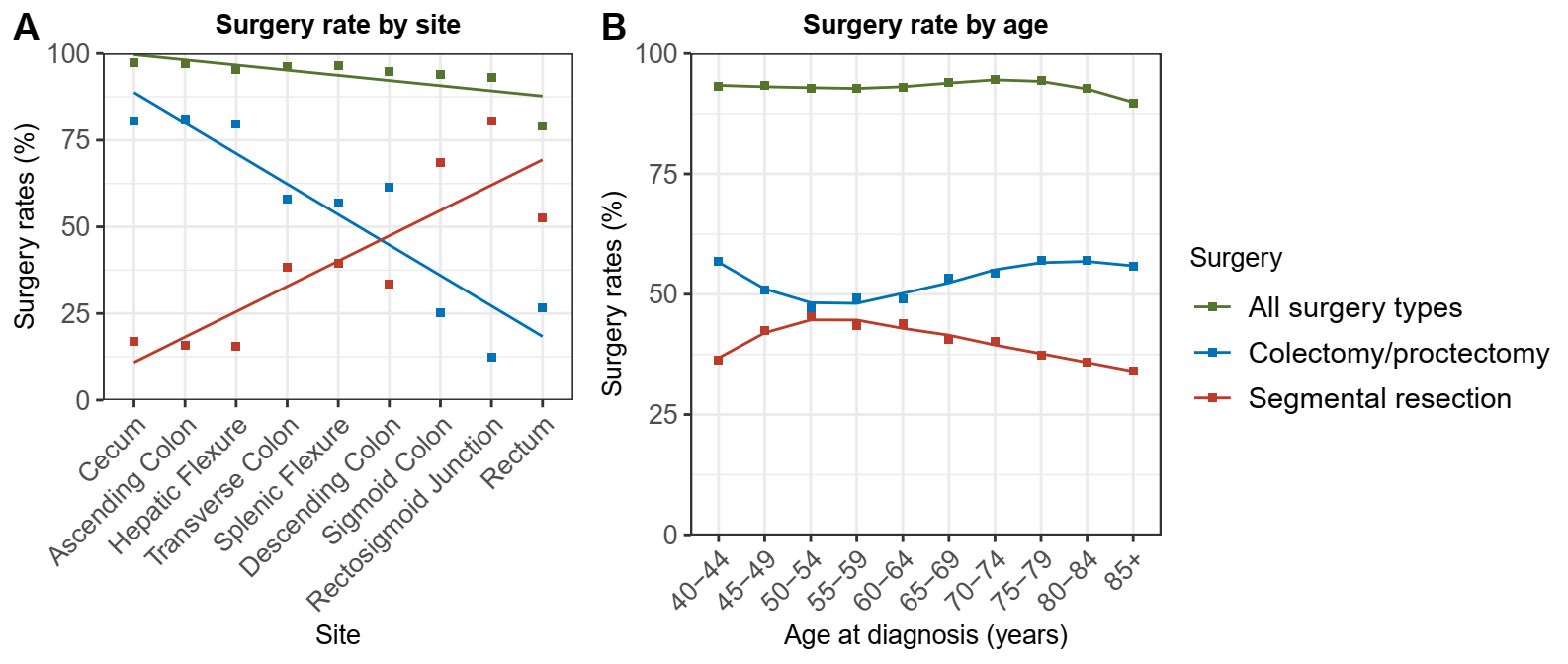


**Figure S1.** Trends of surgical rates by anatomic sites and age. (A) Trends of surgical rates by anatomic sites. (B) Trends of surgical rates by age.


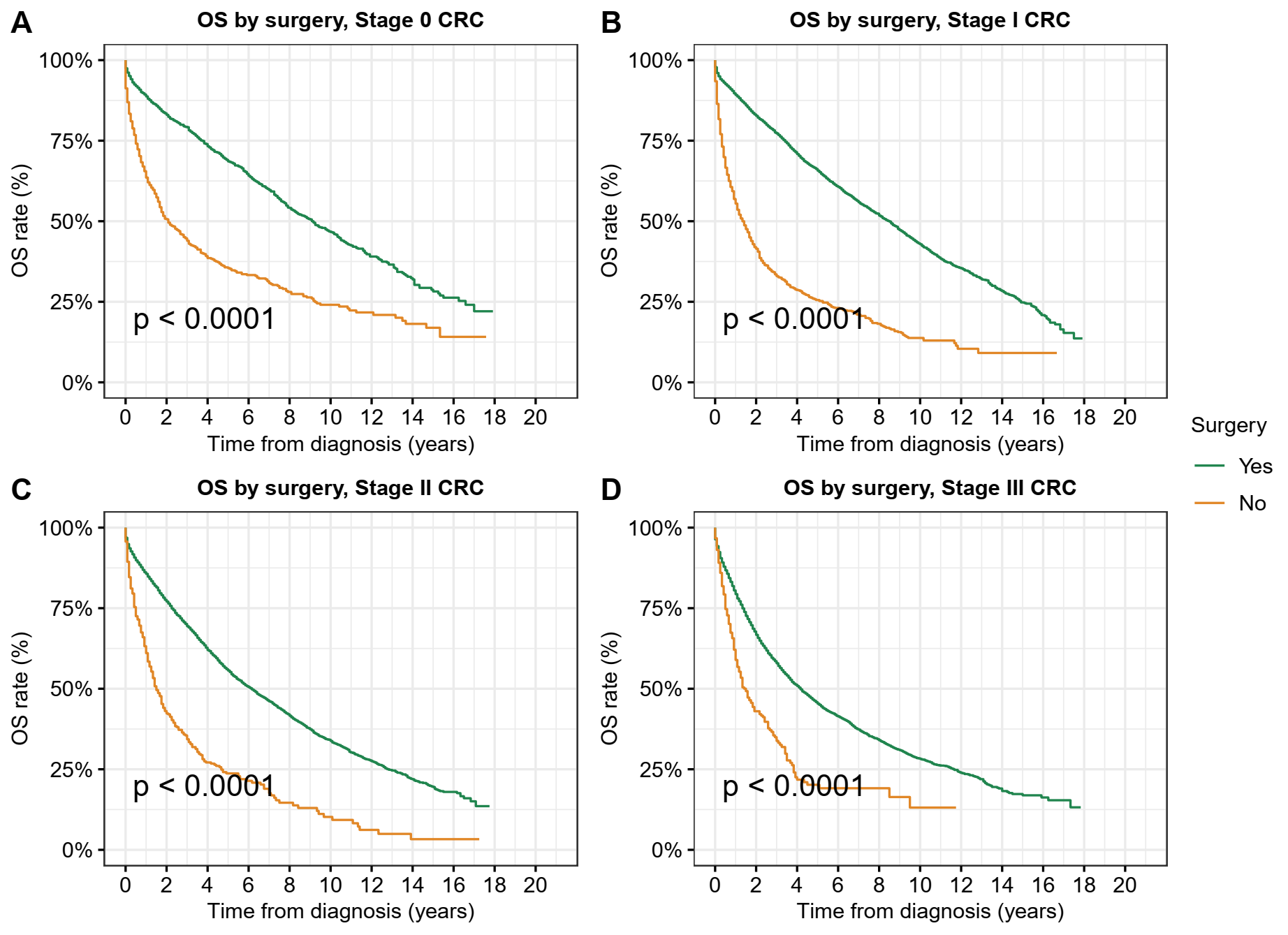


**Figure S2.** Overall survival (OS) of patients with second primary CRC by surgery and tumor stage. OS of patients with (A) stage 0, (B) stage I, (C) stage II, (D) stage III second primary CRC by surgery.


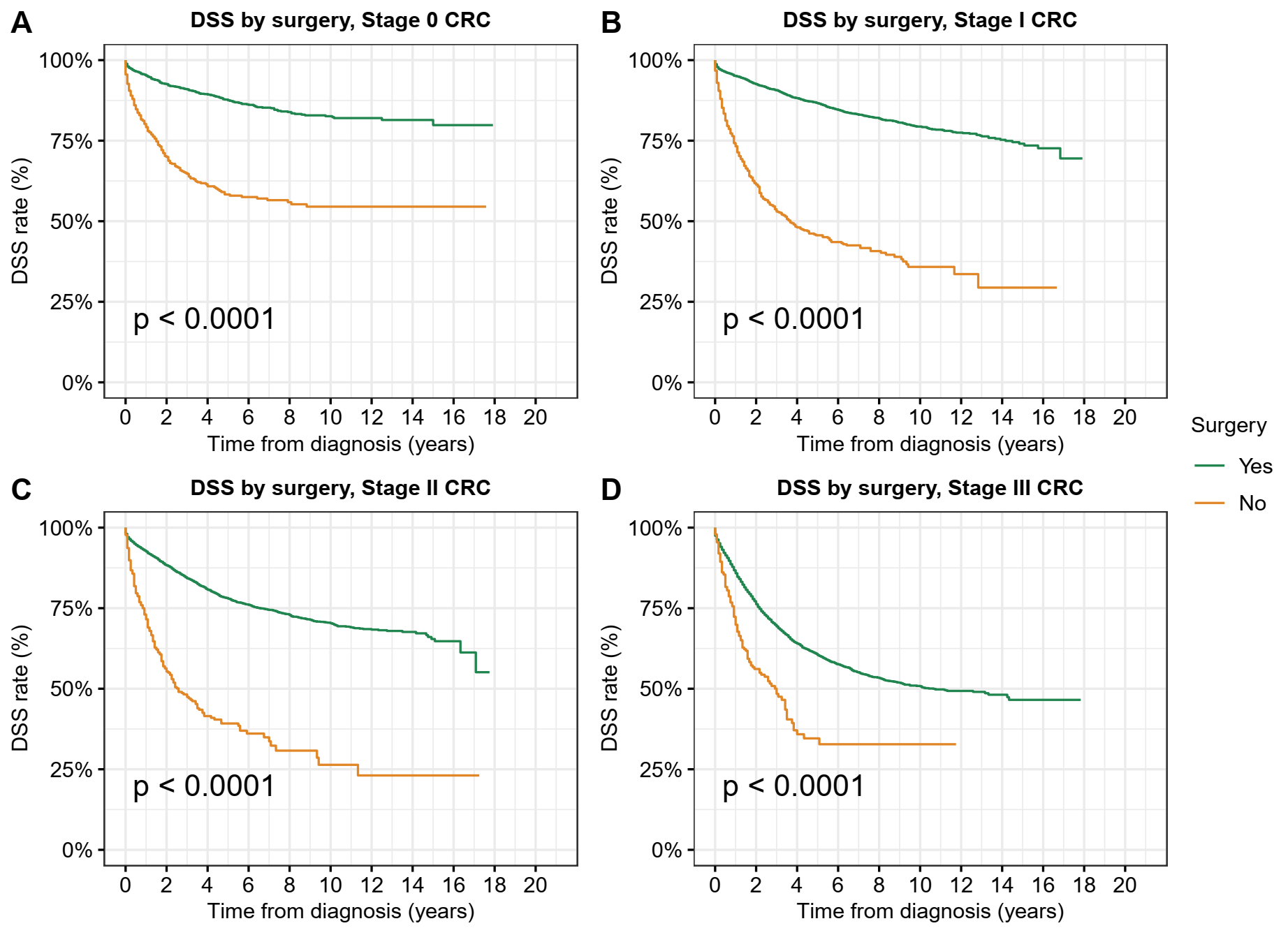


**Figure S3.** Disease-specific survival (DSS) of patients with second primary CRC by surgery and tumor stage. DSS of patients with (A) stage 0, (B) stage I, (C) stage II, (D) stage III second primary CRC by surgery.


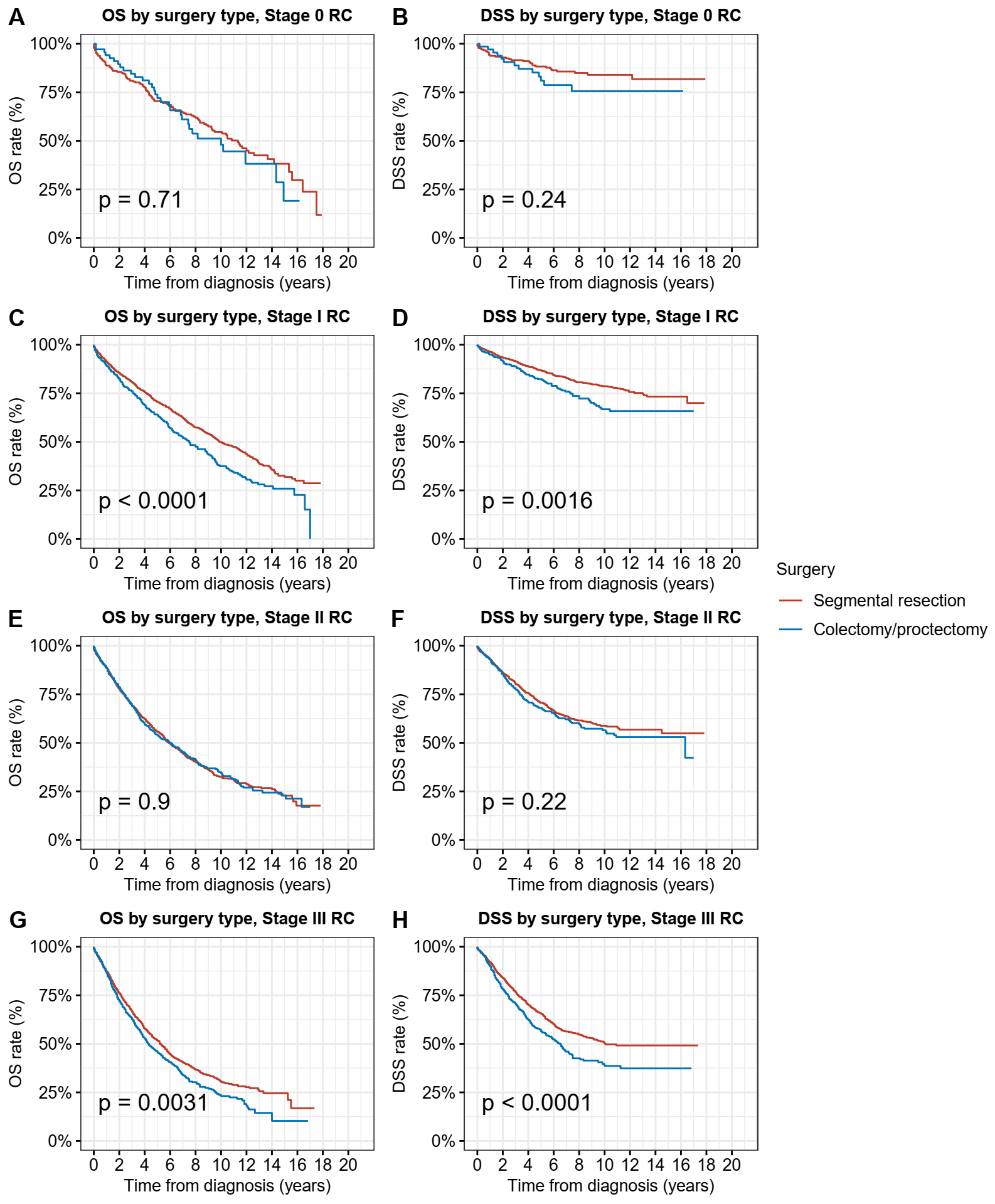


**Figure S4.** Overall survival (OS) and disease-specific survival (DSS) of patients with second primary RC by types of surgical procedures and tumor stage. (A) OS and (B) DSS of patients with stage 0 second primary RC by types of surgical procedures. (C) OS and (D) DSS of patients with stage I second primary RC by types of surgical procedures. (E) OS and (F) DSS of patients with stage II second primary RC by types of surgical procedures. (G) OS and (H) DSS of patients with stage III second primary RC by types of surgical procedures.


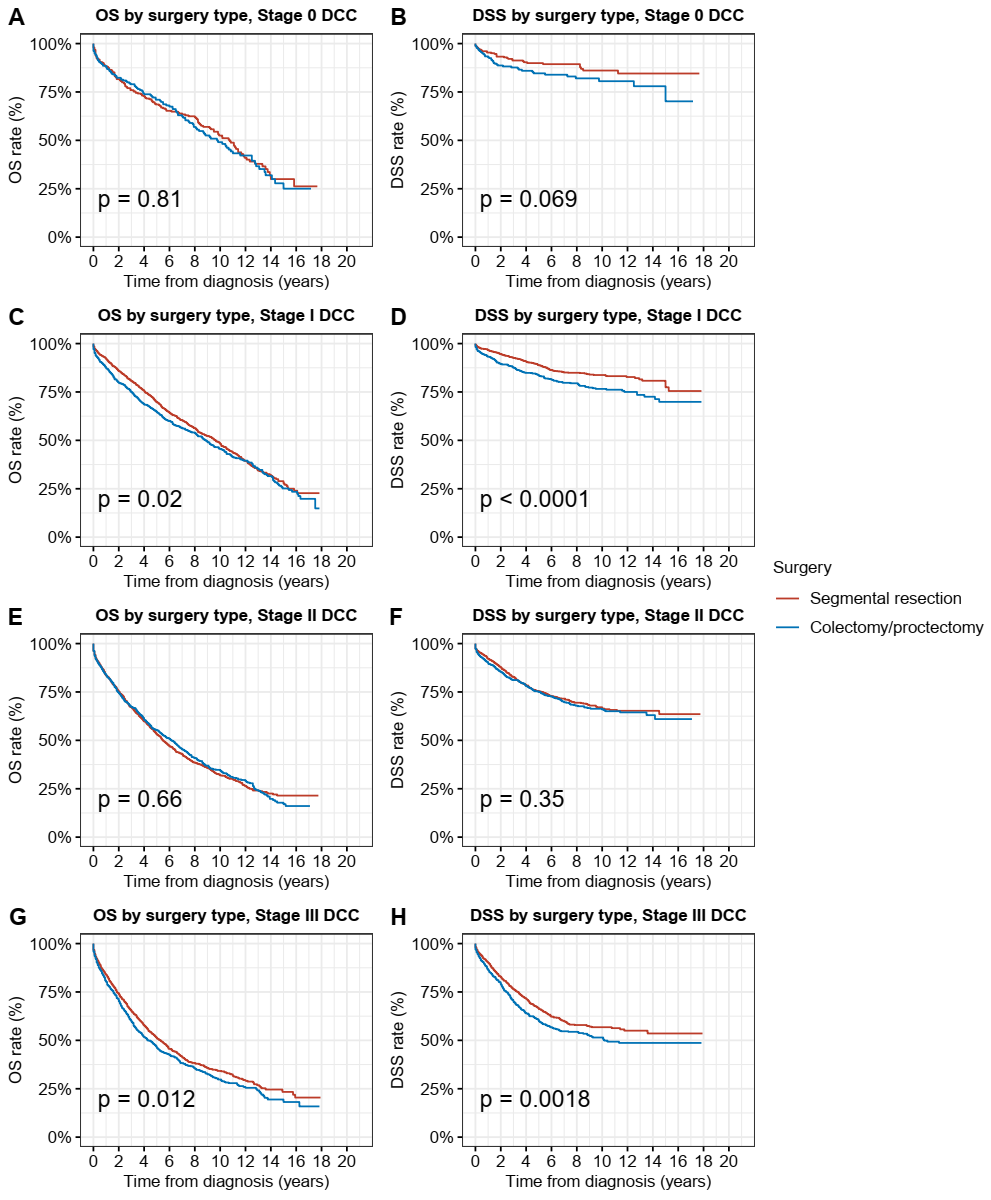


**Figure S5.** Overall survival (OS) and disease-specific survival (DSS) of patients with second primary DCC by types of surgical procedures and tumor stage. (A) OS and (B) DSS of patients with stage 0 second primary DCC by types of surgical procedures. (C) OS and (D) DSS of patients with stage I second primary DCC by types of surgical procedures. (E) OS and (F) DSS of patients with stage II second primary DCC by types of surgical procedures. (G) OS and (H) DSS of patients with stage III second primary DCC by types of surgical procedures.


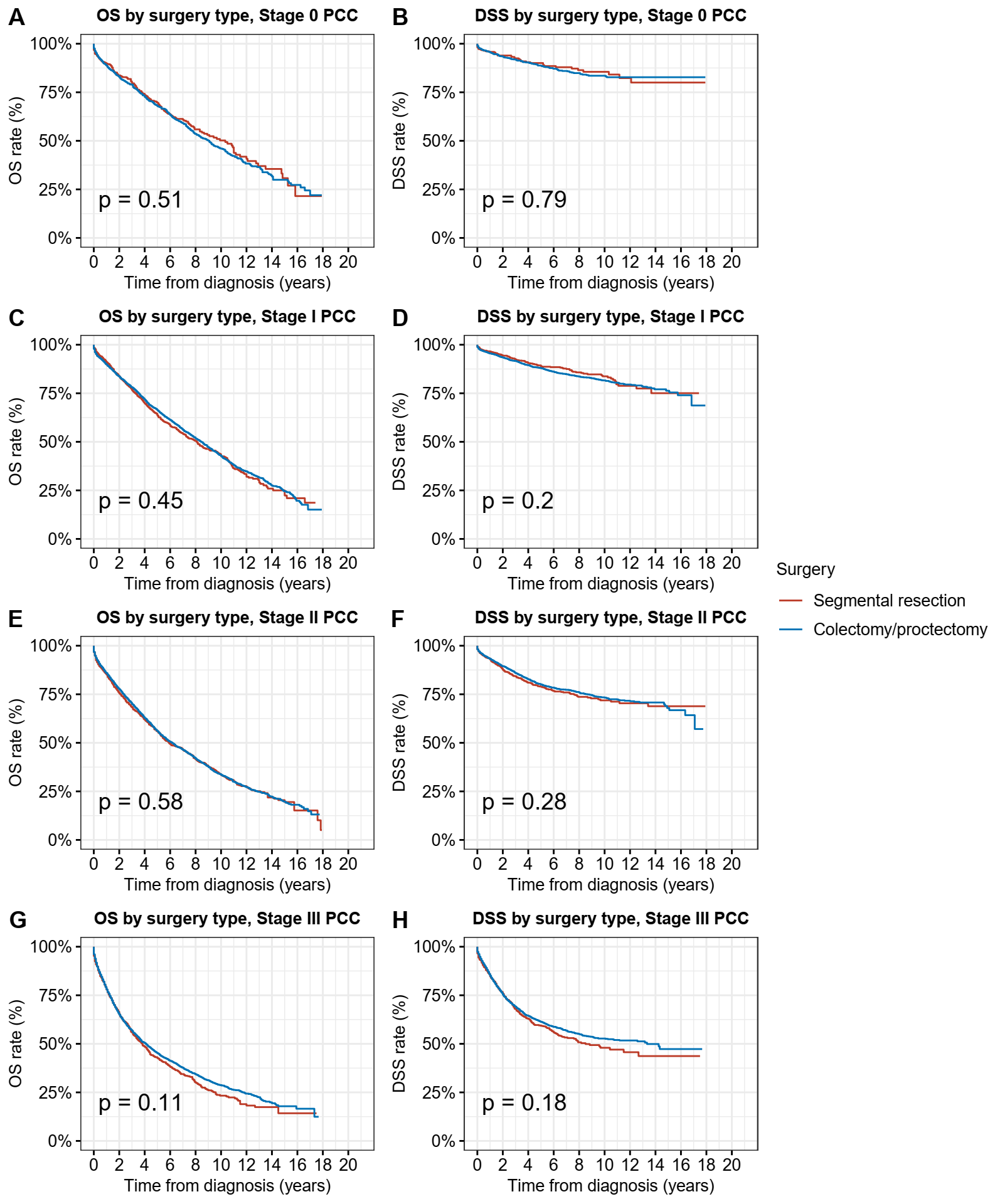


**Figure S6.** Overall survival (OS) and disease-specific survival (DSS) of patients with second primary PCC by types of surgical procedures and tumor stage. (A) OS and (B) DSS of patients with stage 0 second primary PCC by types of surgical procedures. (C) OS and (D) DSS of patients with stage I second primary PCC by types of surgical procedures. (E) OS and (F) DSS of patients with stage II second primary PCC by types of surgical procedures. (G) OS and (H) DSS of patients with stage III second primary PCC by types of surgical procedures.
